# Supplementary material for: Maternal ambient air pollution exposure and risk of stillbirth in Georgia, USA
Source: medRxiv. 2026 Jan 27:2026.01.26.26344822. Preprint. [Version 1] doi: 10.64898/2026.01.26.26344822 (PMC12870622; doi:10.64898/2026.01.26.26344822)

**Supplementary Material**

**Table S1**. Pearson Correlation Coefficients matrix between weekly levels of all ten pollutants from 2005 to 2014.

|  | CO | NO3 | NOx | O3 | PM10 | PM2.5 | EC | OC | NO3 | NH3 |
| --- | --- | --- | --- | --- | --- | --- | --- | --- | --- | --- |
| CO | 1 | 0.812 | 0.786 | -0.028 | 0.397 | 0.509 | 0.744 | 0.551 | 0.433 | 0.481 |
| NO2 | 0.812 | 1 | 0.888 | -0.01 | 0.314 | 0.407 | 0.762 | 0.358 | 0.47 | 0.358 |
| NOx | 0.786 | 0.888 | 1 | -0.211 | 0.184 | 0.273 | 0.752 | 0.29 | 0.48 | 0.211 |
| O3 | -0.028 | -0.01 | -0.211 | 1 | 0.589 | 0.56 | 0.082 | 0.245 | -0.352 | 0.393 |
| PM10 | 0.397 | 0.314 | 0.184 | 0.589 | 1 | 0.817 | 0.444 | 0.527 | -0.039 | 0.645 |
| PM2.5 | 0.509 | 0.407 | 0.273 | 0.56 | 0.817 | 1 | 0.542 | 0.671 | 0.148 | 0.798 |
| EC | 0.744 | 0.762 | 0.752 | 0.082 | 0.444 | 0.542 | 1 | 0.531 | 0.363 | 0.384 |
| OC | 0.551 | 0.358 | 0.29 | 0.245 | 0.527 | 0.671 | 0.531 | 1 | 0.3 | 0.604 |
| NO3 | 0.433 | 0.47 | 0.48 | -0.352 | -0.039 | 0.148 | 0.363 | 0.3 | 1 | 0.315 |
| NH4 | 0.481 | 0.358 | 0.211 | 0.393 | 0.645 | 0.798 | 0.384 | 0.604 | 0.315 | 1 |

**Table S2**. Pearson Correlation Coefficients between different exposure windows within each of ten air pollutants.

| Pollutants | Cor (T1, T2) | Cor (T1, T3) | Cor (T2, T3) |
| --- | --- | --- | --- |
| CO | 0.9414 | -0.0008 | -0.0019 |
| NO2 | 0.9166 | -0.0007 | -0.0031 |
| NOx | 0.8034 | 0.0006 | 0.0005 |
| O3 | 0.0647 | -0.0034 | -0.0028 |
| PM10 | 0.4445 | 0.0022 | -0.0012 |
| PM2.5 | 0.631 | 0.0022 | 0.0033 |
| EC | 0.8264 | 0.0047 | 0.0064 |
| OC | 0.8841 | 0.0034 | 0.0042 |
| NO3 | 0.3709 | -0.0016 | -0.0081 |
| NH4 | 0.8104 | 0.0022 | 0.0045 |

We also constructed the same table for stillbirths with gestational age >=30 weeks. Because many stillbirths have a short third trimester, correlations between third trimester exposure and earlier trimesters can seem low. Restricting to later stillbirths reduces can give a clearer depiction of trimester correlations.

| Pollutants | Cor (T1, T2) | Cor (T1, T3) | Cor (T2, T3) |
| --- | --- | --- | --- |
| CO | 0.9407 | 0.8974 | 0.9421 |
| NO2 | 0.9157 | 0.8572 | 0.9247 |
| NOx | 0.7995 | 0.6422 | 0.8128 |
| O3 | 0.0337 | -0.6803 | 0.1081 |
| PM10 | 0.431 | -0.0064 | 0.4763 |
| PM2.5 | 0.6257 | 0.3857 | 0.6451 |
| EC | 0.8266 | 0.7208 | 0.8375 |
| OC | 0.8857 | 0.8307 | 0.8868 |
| NO3 | 0.3544 | -0.1123 | 0.4154 |
| NH4 | 0.8082 | 0.6802 | 0.8206 |

**Table S3**. Hazard ratio (HR) estimates per unit IQR increase and 95% CI's for all ten pollutants during five exposure windows (the first month, weekly exposure, the first trimester exposure, the second trimester exposure, and the third trimester exposure) for all stillbirths, stillbirths that happened in the second trimester, and stillbirths that happened in the third trimester.

| Exposure time window | Pollutants | All stillbirth (n=8384) | 2nd trimester (4670) | 3rd trimester (3714) |
| --- | --- | --- | --- | --- |
| The first month | CO | 1.018 (0.957, 1.082) | 1.074 (0.974, 1.185) | 0.938 (0.852, 1.032) |
|  | NO2 | 0.973 (0.938, 1.009) | 1.019 (0.954, 1.088) | 0.908 (0.845, 0.977) |
|  | NOx | 0.985 (0.955, 1.017) | 0.996 (0.952, 1.041) | 0.966 (0.920, 1.015) |
|  | O3 | 0.959 (0.909, 1.012) | 0.956 (0.873, 1.048) | 0.961 (0.862, 1.072) |
|  | PM10 | 0.993 (0.966, 1.020) | 1.033 (0.976, 1.092) | 0.935 (0.879, 0.993) |
|  | PM2.5 | 0.991 (0.964, 1.018) | 1.023 (0.965, 1.085) | 0.948 (0.890, 1.010) |
|  | EC | 0.978 (0.948, 1.009) | 1.012 (0.961, 1.066) | 0.927 (0.854, 1.006) |
|  | OC | 1.006 (0.960, 1.055) | 1.031 (0.959, 1.109) | 0.977 (0.892, 1.070) |
|  | NO3 | 1.004 (0.972, 1.037) | 1.020 (0.969, 1.074) | 0.991 (0.921, 1.066) |
|  | NH4 | 1.003 (0.969, 1.037) | 1.053 (0.972, 1.140) | 0.939 (0.872, 1.010) |
| Weekly | CO | 1.018 (1.006, 1.030) | 1.035 (1.012, 1.057) | 0.995 (0.978, 1.012) |
|  | NO2 | 1.011 (0.999, 1.022) | 1.030 (1.011, 1.049) | 0.985 (0.962, 1.008) |
|  | NOx | 1.007 (0.999, 1.015) | 1.020 (1.006, 1.035) | 0.988 (0.974, 1.002) |
|  | O3 | 1.037 (1.001, 1.074) | 1.046 (0.990, 1.106) | 1.030 (0.992, 1.069) |
|  | PM10 | 1.024 (1.000, 1.048) | 1.045 (1.017, 1.073) | 1.000 (0.961, 1.040) |
|  | PM2.5 | 1.010 (0.985, 1.036) | 1.034 (1.001, 1.069) | 0.983 (0.949, 1.019) |
|  | EC | 1.012 (0.992, 1.033) | 1.029 (1.001, 1.058) | 0.993 (0.963, 1.024) |
|  | OC | 1.007 (0.988, 1.026) | 1.015 (0.993, 1.038) | 0.995 (0.971, 1.021) |
|  | NO3 | 1.006 (0.988, 1.024) | 1.024 (0.999, 1.050) | 0.987 (0.959, 1.016) |
|  | NH4 | 1.009 (0.992, 1.027) | 1.025 (1.003, 1.048) | 0.994 (0.968, 1.020) |
| The first trimester cumulative average | CO | 1.060 (0.981, 1.144) | 1.105 (0.979, 1.247) | 0.998 (0.891, 1.118) |
|  | NO2 | 1.002 (0.947, 1.059) | 1.050 (0.958, 1.151) | 0.939 (0.861, 1.025) |
|  | NOx | 1.002 (0.953, 1.053) | 1.024 (0.956, 1.097) | 0.965 (0.893, 1.042) |
|  | O3 | 0.974 (0.924, 1.026) | 0.937 (0.842, 1.042) | 1.030 (0.905, 1.173) |
|  | PM10 | 1.000 (0.959, 1.043) | 1.039 (0.951, 1.136) | 0.943 (0.851, 1.045) |
|  | PM2.5 | 1.002 (0.975, 1.030) | 1.037 (0.970, 1.110) | 0.954 (0.879, 1.036) |
|  | EC | 0.993 (0.937, 1.052) | 1.026 (0.932, 1.130) | 0.948 (0.868, 1.035) |
|  | OC | 1.037 (0.984, 1.094) | 1.035 (0.940, 1.141) | 1.043 (0.936, 1.162) |
|  | NO3 | 1.008 (0.972, 1.045) | 1.069 (1.004, 1.139) | 0.934 (0.857, 1.018) |
|  | NH4 | 1.013 (0.984, 1.043) | 1.072 (0.986, 1.166) | 0.940 (0.857, 1.031) |
| The second trimester cumulative average | CO | 1.079 (0.976, 1.193) | 1.126 (0.993, 1.276) | 0.979 (0.819, 1.170) |
|  | NO2 | 1.064 (1.002, 1.131) | 1.121 (1.033, 1.215) | 0.963 (0.845, 1.097) |
|  | NOx | 1.026 (0.983, 1.070) | 1.084 (0.998, 1.178) | 0.917 (0.791, 1.064) |
|  | O3 | 1.049 (0.972, 1.131) | 1.062 (0.975, 1.156) | 1.035 (0.921, 1.163) |
|  | PM10 | 1.073 (1.041, 1.106) | 1.089 (1.045, 1.136) | 1.033 (0.977, 1.091) |
|  | PM2.5 | 1.045 (1.006, 1.085) | 1.067 (1.011, 1.125) | 0.997 (0.933, 1.066) |
|  | EC | 1.045 (0.997, 1.096) | 1.078 (1.001, 1.160) | 0.987 (0.891, 1.093) |
|  | OC | 1.024 (0.967, 1.083) | 1.090 (1.011, 1.175) | 0.909 (0.813, 1.017) |
|  | NO3 | 1.016 (0.962, 1.072) | 1.089 (1.015, 1.169) | 0.890 (0.803, 0.985) |
|  | NH4 | 1.058 (1.022, 1.095) | 1.065 (1.016, 1.116) | 1.041 (0.956, 1.134) |
| The third trimester cumulative average | CO | - | - | 1.001 (0.992, 1.010) |
|  | NO2 | - | - | 1.000 (0.990, 1.010) |
|  | NOx | - | - | 0.997 (0.991, 1.003) |
|  | O3 | - | - | 1.023 (1.002, 1.044) |
|  | PM10 | - | - | 1.000 (0.991, 1.009) |
|  | PM2.5 | - | - | 0.998 (0.985, 1.010) |
|  | EC | - | - | 1.000 (0.989, 1.010) |
|  | OC | - | - | 0.996 (0.984, 1.008) |
|  | NO3 | - | - | 0.995 (0.984, 1.007) |
|  | NH4 | - | - | 0.995 (0.983, 1.008) |

**Table S4**. Hazard ratio (HR) estimates per unit IQR increase and 95% CI's for all ten pollutants during five exposure windows (the first month, weekly exposure, the first trimester exposure, the second trimester exposure, and the third trimester exposure) for stillbirths that happened after the 37^th^ week of pregnancy.

| Exposure time window | Pollutants | Stillbirth after the 37^th^ week |
| --- | --- | --- |
| The first month | CO | 0.860 (0.682, 1.085) |
|  | NO2 | 0.879 (0.702, 1.100) |
|  | NOx | 0.949 (0.794, 1.134) |
|  | O3 | 0.962 (0.814, 1.138) |
|  | PM10 | 0.930 (0.811, 1.066) |
|  | PM2.5 | 0.953 (0.850, 1.068) |
|  | EC | 0.831 (0.647, 1.067) |
|  | OC | 0.942 (0.777, 1.143) |
|  | NO3 | 0.934 (0.828, 1.054) |
|  | NH4 | 0.998 (0.873, 1.141) |
| Weekly | CO | 1.068 (0.896, 1.273) |
|  | NO2 | 1.029 (0.839, 1.261) |
|  | NOx | 1.028 (0.937, 1.129) |
|  | O3 | 1.122 (0.947, 1.328) |
|  | PM10 | 1.019 (0.917, 1.133) |
|  | PM2.5 | 1.042 (0.931, 1.167) |
|  | EC | 1.032 (0.939, 1.134) |
|  | OC | 0.972 (0.863, 1.094) |
|  | NO3 | 0.977 (0.880, 1.085) |
|  | NH4 | 0.988 (0.892, 1.095) |
| The first trimester cumulative average | CO | 0.933 (0.731, 1.190) |
|  | NO2 | 0.942 (0.744, 1.192) |
|  | NOx | 1.005 (0.859, 1.175) |
|  | O3 | 1.060 (0.813, 1.383) |
|  | PM10 | 1.146 (0.874, 1.503) |
|  | PM2.5 | 1.087 (0.874, 1.353) |
|  | EC | 0.884 (0.712, 1.099) |
|  | OC | 0.938 (0.703, 1.253) |
|  | NO3 | 0.776 (0.643, 0.936) |
|  | NH4 | 1.034 (0.773, 1.383) |
| The second trimester cumulative average | CO | 0.838 (0.513, 1.369) |
|  | NO2 | 1.552 (1.193, 2.019) |
|  | NOx | 1.104 (0.894, 1.362) |
|  | O3 | 1.026 (0.739, 1.424) |
|  | PM10 | 1.062 (0.858, 1.313) |
|  | PM2.5 | 0.966 (0.799, 1.168) |
|  | EC | 1.118 (0.925, 1.350) |
|  | OC | 0.800 (0.547, 1.172) |
|  | NO3 | 0.867 (0.694, 1.082) |
|  | NH4 | 1.011 (0.840, 1.218) |
| The third trimester cumulative average | CO | 0.728 (0.373, 1.422) |
|  | NO2 | 0.767 (0.471, 1.250) |
|  | NOx | 0.756 (0.511, 1.118) |
|  | O3 | 1.349 (0.815, 2.232) |
|  | PM10 | 1.135 (0.975, 1.321) |
|  | PM2.5 | 1.055 (0.850, 1.309) |
|  | EC | 0.977 (0.668, 1.429) |
|  | OC | 0.889 (0.629, 1.256) |
|  | NO3 | 1.209 (0.946, 1.545) |
|  | NH4 | 1.127 (0.872, 1.457) |

**Table S5**. Hazard ratio (HR) estimates per unit IQR increase and 95% CI's for all ten pollutants during five exposure windows (the first month, weekly exposure, the first trimester exposure, the second trimester exposure, and the third trimester exposure) for all stillbirths, stillbirths that happened in the second trimester, and stillbirths that happened in the third trimester, adjusting for weekly average maximum temperature.

| Exposure time window | Pollutants | All stillbirth (n=8384) | 2nd trimester (4670) | 3rd trimester (3714) |
| --- | --- | --- | --- | --- |
| The first month | CO | 1.011 (0.952, 1.074) | 1.066 (0.968, 1.175) | 0.933 (0.850, 1.024) |
|  | NO2 | 0.974 (0.938, 1.010) | 1.020 (0.954, 1.091) | 0.909 (0.846, 0.977) |
|  | NOx | 0.985 (0.955, 1.016) | 0.995 (0.952, 1.040) | 0.966 (0.920, 1.015) |
|  | O3 | 0.941 (0.888, 0.997) | 0.934 (0.844, 1.033) | 0.948 (0.854, 1.052) |
|  | PM10 | 0.980 (0.952, 1.009) | 1.019 (0.966, 1.076) | 0.923 (0.865, 0.984) |
|  | PM2.5 | 0.982 (0.953, 1.011) | 1.013 (0.951, 1.078) | 0.941 (0.884, 1.000) |
|  | EC | 0.970 (0.942, 1.000) | 1.003 (0.951, 1.057) | 0.921 (0.850, 0.997) |
|  | OC | 0.997 (0.949, 1.047) | 1.020 (0.944, 1.102) | 0.970 (0.884, 1.064) |
|  | NO3 | 1.019 (0.986, 1.052) | 1.041 (0.981, 1.104) | 1.000 (0.927, 1.079) |
|  | NH4 | 1.004 (0.970, 1.038) | 1.054 (0.973, 1.142) | 0.940 (0.873, 1.011) |
| Weekly | CO | 1.076 (1.024, 1.131) | 1.152 (1.054, 1.260) | 0.978 (0.912, 1.050) |
|  | NO2 | 1.039 (0.998, 1.081) | 1.112 (1.042, 1.188) | 0.947 (0.872, 1.028) |
|  | NOx | 1.024 (0.995, 1.054) | 1.072 (1.019, 1.128) | 0.959 (0.914, 1.007) |
|  | O3 | 1.059 (0.999, 1.122) | 1.076 (0.983, 1.178) | 1.046 (0.981, 1.115) |
|  | PM10 | 1.039 (0.996, 1.084) | 1.076 (1.028, 1.127) | 0.997 (0.929, 1.069) |
|  | PM2.5 | 1.016 (0.974, 1.058) | 1.056 (1.001, 1.115) | 0.972 (0.916, 1.031) |
|  | EC | 1.028 (0.980, 1.078) | 1.069 (1.002, 1.141) | 0.983 (0.914, 1.058) |
|  | OC | 1.018 (0.964, 1.075) | 1.043 (0.980, 1.111) | 0.986 (0.917, 1.060) |
|  | NO3 | 1.016 (0.982, 1.050) | 1.050 (1.003, 1.100) | 0.979 (0.922, 1.040) |
|  | NH4 | 1.023 (0.982, 1.065) | 1.060 (1.007, 1.116) | 0.987 (0.929, 1.048) |
| The first trimester cumulative average | CO | 1.058 (0.980, 1.142) | 1.103 (0.978, 1.245) | 0.995 (0.889, 1.114) |
|  | NO2 | 1.004 (0.949, 1.063) | 1.052 (0.958, 1.157) | 0.942 (0.864, 1.028) |
|  | NOx | 1.003 (0.954, 1.055) | 1.026 (0.956, 1.100) | 0.967 (0.895, 1.046) |
|  | O3 | 0.962 (0.911, 1.016) | 0.926 (0.830, 1.033) | 1.017 (0.897, 1.153) |
|  | PM10 | 0.992 (0.953, 1.033) | 1.036 (0.952, 1.127) | 0.930 (0.838, 1.032) |
|  | PM2.5 | 0.997 (0.971, 1.025) | 1.035 (0.968, 1.105) | 0.947 (0.875, 1.024) |
|  | EC | 0.990 (0.934, 1.050) | 1.024 (0.930, 1.128) | 0.944 (0.862, 1.033) |
|  | OC | 1.033 (0.976, 1.092) | 1.032 (0.932, 1.142) | 1.037 (0.927, 1.159) |
|  | NO3 | 1.022 (0.986, 1.059) | 1.088 (1.020, 1.161) | 0.944 (0.861, 1.034) |
|  | NH4 | 1.014 (0.985, 1.045) | 1.073 (0.986, 1.168) | 0.942 (0.857, 1.035) |
| The second trimester cumulative average | CO | 1.077 (0.975, 1.189) | 1.126 (0.995, 1.274) | 0.975 (0.816, 1.166) |
|  | NO2 | 1.064 (1.001, 1.131) | 1.120 (1.033, 1.215) | 0.963 (0.845, 1.098) |
|  | NOx | 1.026 (0.983, 1.070) | 1.084 (0.998, 1.178) | 0.918 (0.794, 1.062) |
|  | O3 | 1.047 (0.969, 1.133) | 1.063 (0.973, 1.162) | 1.030 (0.918, 1.157) |
|  | PM10 | 1.074 (1.039, 1.109) | 1.092 (1.046, 1.141) | 1.030 (0.972, 1.091) |
|  | PM2.5 | 1.045 (1.006, 1.085) | 1.068 (1.011, 1.128) | 0.994 (0.932, 1.060) |
|  | EC | 1.044 (0.995, 1.096) | 1.079 (1.002, 1.161) | 0.984 (0.882, 1.097) |
|  | OC | 1.022 (0.967, 1.081) | 1.091 (1.012, 1.177) | 0.907 (0.812, 1.013) |
|  | NO3 | 1.019 (0.966, 1.075) | 1.093 (1.011, 1.182) | 0.892 (0.806, 0.988) |
|  | NH4 | 1.058 (1.022, 1.095) | 1.065 (1.016, 1.116) | 1.040 (0.956, 1.132) |
| The third trimester cumulative average | CO | - | - | 1.006 (0.875, 1.156) |
|  | NO2 | - | - | 1.003 (0.873, 1.152) |
|  | NOx | - | - | 0.958 (0.887, 1.035) |
|  | O3 | - | - | 1.119 (1.007, 1.243) |
|  | PM10 | - | - | 0.996 (0.948, 1.046) |
|  | PM2.5 | - | - | 0.984 (0.921, 1.051) |
|  | EC | - | - | 0.993 (0.907, 1.087) |
|  | OC | - | - | 0.959 (0.846, 1.087) |
|  | NO3 | - | - | 0.977 (0.896, 1.066) |
|  | NH4 | - | - | 0.963 (0.870, 1.066) |

**Table S6**. Hazard ratio (HR) estimates per unit IQR increase and 95% CI's for all ten pollutants during five exposure windows (the first month, weekly exposure, the first trimester exposure, the second trimester exposure, and the third trimester exposure) for all stillbirths, stillbirths that happened in the second trimester, and stillbirths that happened in the third trimester, adjusting for weekly average minimum temperature.

| Exposure time window | Pollutants | All stillbirth (n=8384) | 2nd trimester (4670) | 3rd trimester (3714) |
| --- | --- | --- | --- | --- |
| The first month | CO | 1.018 (0.957, 1.082) | 1.074 (0.974, 1.185) | 0.938 (0.852, 1.032) |
|  | NO2 | 0.973 (0.938, 1.009) | 1.019 (0.954, 1.088) | 0.908 (0.845, 0.977) |
|  | NOx | 0.985 (0.955, 1.017) | 0.996 (0.952, 1.041) | 0.966 (0.920, 1.015) |
|  | O3 | 0.959 (0.909, 1.012) | 0.956 (0.873, 1.048) | 0.961 (0.862, 1.072) |
|  | PM10 | 0.993 (0.966, 1.020) | 1.033 (0.976, 1.092) | 0.935 (0.879, 0.993) |
|  | PM2.5 | 0.991 (0.964, 1.018) | 1.023 (0.965, 1.085) | 0.948 (0.890, 1.010) |
|  | EC | 0.978 (0.948, 1.009) | 1.012 (0.961, 1.066) | 0.927 (0.854, 1.006) |
|  | OC | 1.006 (0.960, 1.055) | 1.031 (0.959, 1.109) | 0.977 (0.892, 1.070) |
|  | NO3 | 1.004 (0.972, 1.037) | 1.020 (0.969, 1.074) | 0.991 (0.921, 1.066) |
|  | NH4 | 1.003 (0.969, 1.037) | 1.053 (0.972, 1.140) | 0.939 (0.872, 1.010) |
| Weekly | CO | 1.078 (1.022, 1.136) | 1.159 (1.058, 1.269) | 0.974 (0.907, 1.046) |
|  | NO2 | 1.040 (0.996, 1.085) | 1.125 (1.052, 1.202) | 0.935 (0.861, 1.016) |
|  | NOx | 1.025 (0.995, 1.056) | 1.080 (1.026, 1.137) | 0.952 (0.908, 0.998) |
|  | O3 | 1.060 (1.000, 1.123) | 1.079 (0.985, 1.182) | 1.046 (0.982, 1.113) |
|  | PM10 | 1.040 (1.000, 1.081) | 1.076 (1.030, 1.124) | 0.998 (0.933, 1.066) |
|  | PM2.5 | 1.017 (0.976, 1.059) | 1.061 (1.005, 1.119) | 0.969 (0.913, 1.027) |
|  | EC | 1.029 (0.982, 1.079) | 1.076 (1.010, 1.147) | 0.979 (0.911, 1.052) |
|  | OC | 1.019 (0.965, 1.076) | 1.048 (0.984, 1.117) | 0.981 (0.915, 1.052) |
|  | NO3 | 1.013 (0.980, 1.047) | 1.059 (1.008, 1.112) | 0.965 (0.909, 1.024) |
|  | NH4 | 1.022 (0.981, 1.065) | 1.063 (1.009, 1.121) | 0.982 (0.922, 1.045) |
| The first trimester cumulative average | CO | 1.059 (0.981, 1.144) | 1.104 (0.978, 1.247) | 0.998 (0.891, 1.118) |
|  | NO2 | 0.999 (0.941, 1.060) | 1.046 (0.948, 1.155) | 0.938 (0.857, 1.027) |
|  | NOx | 0.999 (0.945, 1.057) | 1.021 (0.945, 1.104) | 0.964 (0.891, 1.044) |
|  | O3 | 0.975 (0.925, 1.029) | 0.939 (0.843, 1.045) | 1.031 (0.907, 1.171) |
|  | PM10 | 1.004 (0.966, 1.043) | 1.047 (0.965, 1.137) | 0.941 (0.850, 1.041) |
|  | PM2.5 | 1.004 (0.977, 1.031) | 1.040 (0.973, 1.111) | 0.954 (0.880, 1.034) |
|  | EC | 0.993 (0.938, 1.052) | 1.027 (0.933, 1.130) | 0.948 (0.867, 1.036) |
|  | OC | 1.039 (0.984, 1.097) | 1.037 (0.940, 1.145) | 1.043 (0.936, 1.163) |
|  | NO3 | 1.000 (0.959, 1.043) | 1.065 (0.991, 1.144) | 0.925 (0.843, 1.014) |
|  | NH4 | 1.012 (0.980, 1.045) | 1.071 (0.983, 1.167) | 0.940 (0.855, 1.033) |
| The second trimester cumulative average | CO | 1.079 (0.976, 1.193) | 1.126 (0.993, 1.276) | 0.979 (0.819, 1.169) |
|  | NO2 | 1.068 (1.005, 1.135) | 1.128 (1.040, 1.224) | 0.963 (0.842, 1.102) |
|  | NOx | 1.028 (0.985, 1.072) | 1.089 (1.003, 1.182) | 0.917 (0.794, 1.060) |
|  | O3 | 1.048 (0.972, 1.130) | 1.061 (0.975, 1.156) | 1.035 (0.922, 1.162) |
|  | PM10 | 1.073 (1.041, 1.106) | 1.088 (1.043, 1.135) | 1.033 (0.977, 1.092) |
|  | PM2.5 | 1.045 (1.006, 1.085) | 1.066 (1.011, 1.125) | 0.997 (0.934, 1.065) |
|  | EC | 1.045 (0.997, 1.097) | 1.078 (1.002, 1.160) | 0.987 (0.890, 1.094) |
|  | OC | 1.024 (0.967, 1.084) | 1.091 (1.012, 1.176) | 0.909 (0.813, 1.017) |
|  | NO3 | 1.019 (0.967, 1.074) | 1.100 (1.014, 1.193) | 0.888 (0.800, 0.985) |
|  | NH4 | 1.059 (1.022, 1.096) | 1.066 (1.017, 1.117) | 1.041 (0.956, 1.134) |
| The third trimester cumulative average | CO | - | - | 1.010 (0.880, 1.160) |
|  | NO2 | - | - | 0.993 (0.863, 1.142) |
|  | NOx | - | - | 0.953 (0.881, 1.031) |
|  | O3 | - | - | 1.123 (1.013, 1.245) |
|  | PM10 | - | - | 1.002 (0.958, 1.049) |
|  | PM2.5 | - | - | 0.989 (0.925, 1.056) |
|  | EC | - | - | 0.995 (0.912, 1.087) |
|  | OC | - | - | 0.960 (0.850, 1.085) |
|  | NO3 | - | - | 0.962 (0.887, 1.043) |
|  | NH4 | - | - | 0.962 (0.868, 1.065) |

**Figure S1**. Hazard ratio (HR) estimates per unit IQR increase and 95% CI's for all ten pollutants during all five exposure windows restricted to stillbirths occurring after the 37^th^ week of pregnancy.


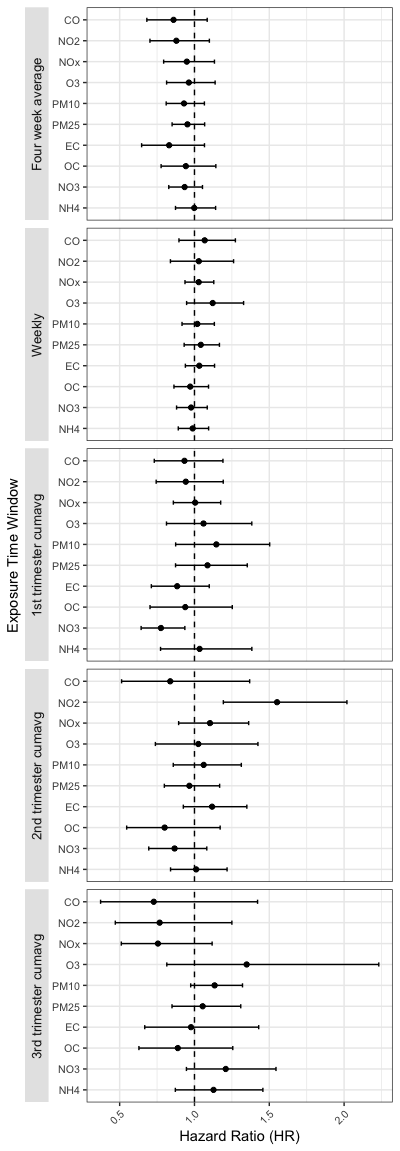


**Figure S2**. Comparison of hazard ratio (HR) estimates per unit IQR increase and 95% CI's for all ten pollutants during all exposure windows (the first week, weekly the first trimester, the second trimester, and the third trimester exposure) for all stillbirths, stillbirths that happened in the second trimester, and stillbirths that happened in the third trimester between main analysis and sensitivity analysis adjusting for weekly average maximum temperature.


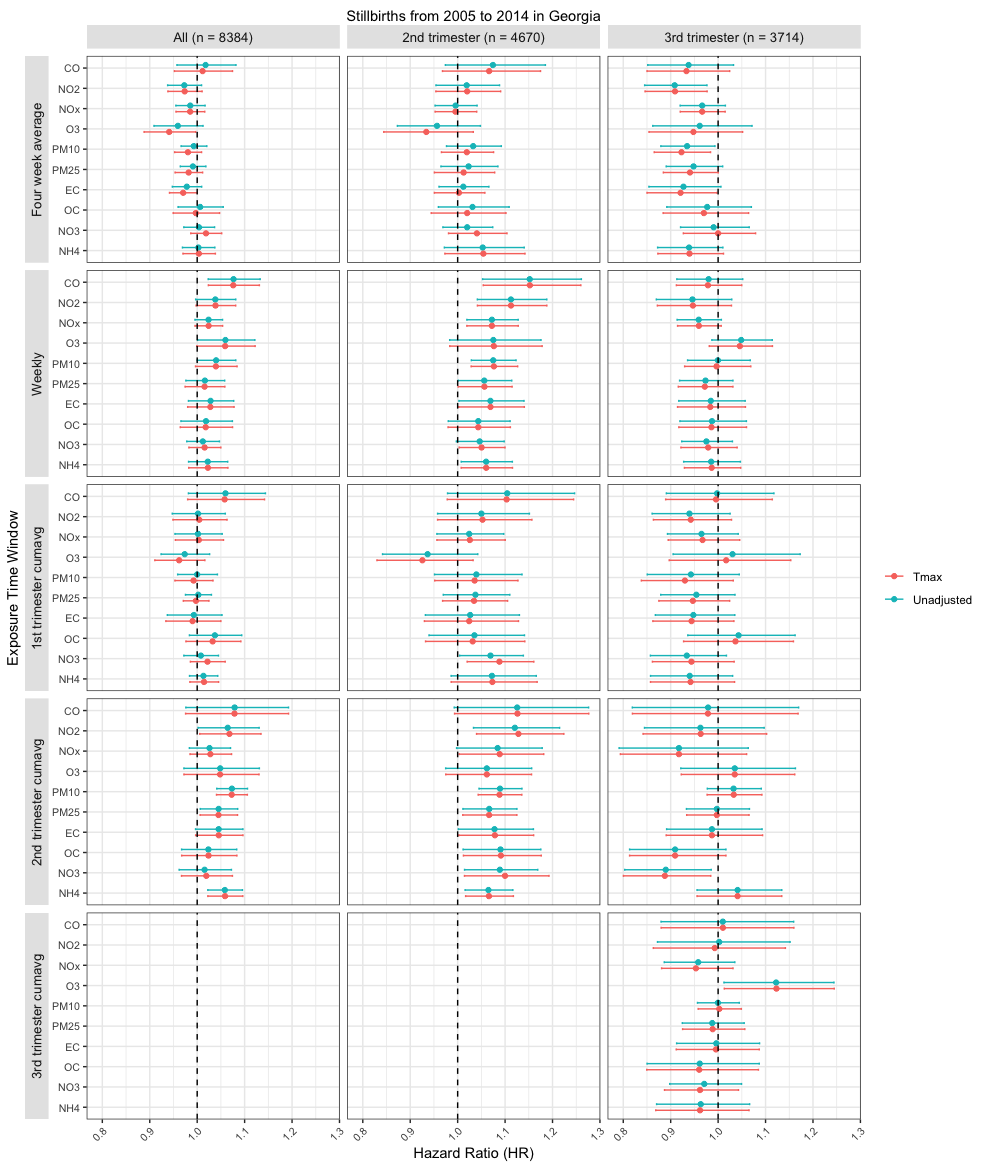


**Figure S3**. Comparison of hazard ratio (HR) estimates per unit IQR increase and 95% CI's for all ten pollutants during all exposure windows (the first week, weekly the first trimester, the second trimester, and the third trimester exposure) for all stillbirths, stillbirths that happened in the second trimester, and stillbirths that happened in the third trimester between main analysis and sensitivity analysis adjusting for weekly average minimum temperature.


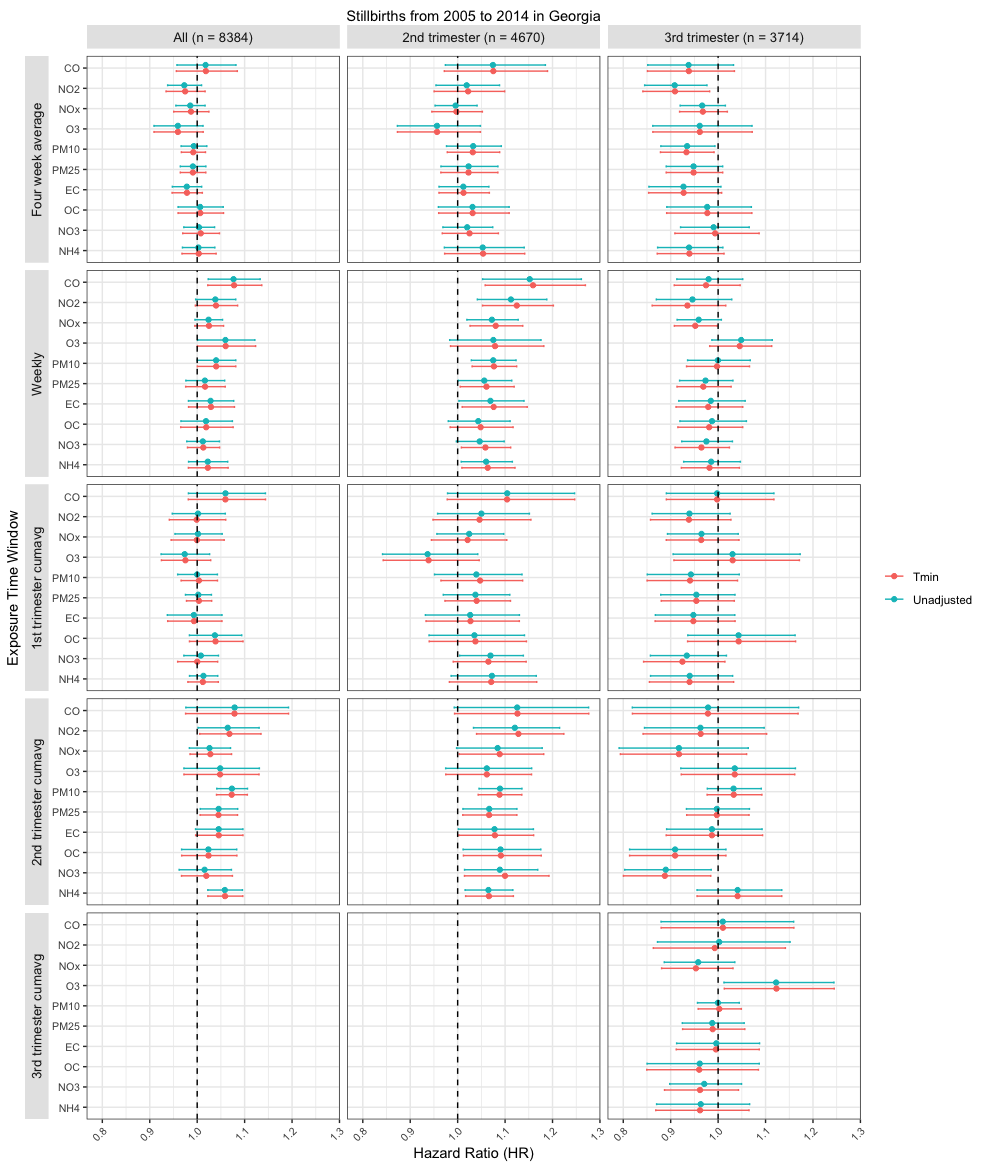

Supplement: Supplement 1 [file media-1.docx]
